# Formalising the induction of patient and public involvement contributors on trial oversight committees

**DOI:** 10.1186/s40900-021-00269-y

**Published:** 2021-06-17

**Authors:** Emily C. Pickering, Bec Hanley, Philip Bell, Jacqui Gath, Patrick Hanlon, Robert Oldroyd, Richard Stephens, Conor D. Tweed

**Affiliations:** 1 AGE Research Unit, Imperial College London, London, UK; 2 Institute of Clinical Trials and Methodology, University College London, London, UK; 3 Medical Research Council Clinical Trials Unit at University College London, London, UK; 4 NCRI Consumer Liaison Group, London, UK

**Keywords:** Patient and Public Involvement, Induction, Oversight committee, Clinical trials, Trial steering committee, Trial management group, Independent data monitoring committee

## Abstract

**Background:**

Clinical Trials Units are encouraged to integrate Patient and Public Involvement (PPI) into all aspects of trial design, running and oversight. This research explored the induction and training of PPI Contributors joining trial oversight committees and was used to update the Medical Research Council Clinical Trials Unit at University College London’s (MRC CTU at UCL) induction pack for new PPI Contributors.

**Methods:**

Published and unpublished materials provided by other CTUs and research organisations on training for PPI Contributors on oversight committees were reviewed, with themes then triangulated to identify the most common topics covered in induction training. A face-to-face workshop with PPI Contributors from the MRC CTU at UCL reviewed a draft updated Induction Pack. Findings from these discussions were incorporated into a revised induction pack which was then re-reviewed by the workshop attendees.

**Results:**

No published literature on this subject was found. However, several common themes were identified from unpublished materials. Workshop attendees agreed with most of the themes suggested in the initial draft pack based on the literature search and also provided a number of additional topics for discussion.

**Conclusions:**

There is very little consistency in the induction of PPI Contributors on oversight committees. Whilst most local guidance explains the general role of a PPI Contributor, more context and background of the particular trial needs to be provided to allow for adequate induction of new committee members. The Induction Pack created provides a framework upon which trial managers can build a full picture of their study.

## Plain English summary

Clinical Trials Units are encouraged to integrate Patient and Public Involvement (PPI) into all aspects of their trials. This research explored the induction and training of PPI Contributors (e.g. patients, carers and members of the public) joining trial oversight committees. These committees regularly review trial progress and ensure participant safety. PPI Contributors sit on the committees to provide important guidance and advice on what the trial is like for a participant taking part.

We looked at existing materials and research to find out what is already in use and how useful PPI Contributors felt it may be. We also held a workshop with existing PPI Contributors who had experience of participating in trials and sitting on oversight committees to find out what information they felt would be useful during the induction of a new member.

Our research was used to create an induction pack for new PPI Contributors at the Medical Research Council Clinical Trials Unit at University College London (MRC CTU at UCL). We have made this resource available to all researchers and in this report we describe how it was created.

## Background

In the United Kingdom, standard practice for clinical trial oversight and management is three-fold in line with European Medicines Agency guidelines [1], being formed of a Trial Steering Committee (TSC), Trial Management Group (TMG) and an Independent Data Monitoring Committee (IDMC) [2]. In recent years, greater importance has been placed on PPI within clinical trials [3–6]. Many funding bodies now insist upon completed and planned PPI activity being detailed in grant applications [7–10], and under the incoming European Union Clinical Trials Regulation any patient or public engagement will have to be documented in study protocols [11]. In addition, the 2018 draft National Standards for Public Involvement from the UK Public Involvement Standards Development Partnership [12], later renamed the UK Standards for Public Involvement, emphasised that PPI best practice involves partnership throughout the life of a study’s design, running and governance. The inclusion of one or more PPI Contributors^1^ on trial oversight groups is one way to address the need for public involvement in trials at the highest level of oversight and accountability. However, sitting on a trial oversight committee requires PPI Contributors to have skills and understanding beyond the experiential contributions that are more common in PPI generally. Contributors to these groups need to feel able to understand and participate in the discussions taking place, and share their valuable insights.

Training materials for PPI Contributors in clinical research have been found to be of poor quality [13] despite evidence showing that involvement is most valuable when Contributors are adequately supported and have clear roles and responsibilities [14]. Clear guidance and better support from the outset of their involvement in a clinical trial is therefore required. The Medical Research Council Clinical Trials Unit at UCL (MRC CTU at UCL) aims to have PPI Contributors on all trial oversight committees as per their committee charters [15]. However, the last induction pack for PPI contributors who join MRC CTU at UCL trials was developed in 2011 and was written for people who get involved in cancer trials. Therefore in late 2017 it was agreed to create a wholly new pack to cover involvement in all of our trial oversight committees. In collaboration with PPI Contributors experienced in trial oversight work, we identified the requisite topics that should be covered in an Induction Pack and during 2018 and 2019 we created an updated resource for the MRC CTU at UCL, encompassing current best practice and research findings.

## Methods

A literature search of published articles detailing the requirements and methods for training and induction of new PPI Contributors was performed. Requests for sharing unpublished induction materials were sent to CTUs and charities with an interest in PPI in research. Key themes and topics found from these materials were then triangulated through identification of repeated concepts and ideas from a variety of sources. These then formed the initial sections of a draft Induction Pack presented to a workshop of existing PPI Contributors from the MRC CTU at UCL who all had experience of participating in research studies and/or sitting on trial oversight committees. A revised draft pack was based on the feedback from the PPI workshop, and utilised adult learning theory and best practice to ensure an optimal presentation and ordering of the information. A second review by the workshop attendees resulted in the completion of a final template.

### Literature search

Based on the known breadth of synonymous terms for PPI and trial oversight committees, a wide-ranging search strategy of Embase, MEDLINE and PsychINFO was employed to identify suitable literature for review. The search strategy employed is listed in Table 1. Inclusion criteria were references to:

1. training or induction materials
2. at least one type trial oversight committee (TMG, TSC or IDMC)
3. PPI Contributors

**Table 1:** Published data search strategy

Criteria Number	Search Criteria
1	Patient represent*
2	Consumer represent*
3	Public represent*
4	Lay Person
5	Lay represent*
6	PPI Contributor
7	1 or 2 or 3 or 4 or 5 or 6
8	Trial oversight group
9	Trial oversight committee
10	Trial steering committee
11	Study steering committee
12	Trial management group
13	Data monitoring committee
14	Data safety and monitoring board
15	8 or 9 or 10 or 11 or 12 or 13 or 14
16	Induction
17	Training
18	Prepar*
19	16 or 17 or 18
20	7 and 15 and 19

PPI leads at CTUs were approached to share local induction materials via the UK Clinical Research Collaboration’s Registered Clinical Trial Units Network (UKCRC CTU). This network of CTUs was selected as these organisations are recognised as experienced centres in clinical trial delivery and design [16]. The network hosts a dedicated PPI sub-group with the specific aim of mapping existing resources and developing collaborative working [17].

Two requests, in April and May 2018, to share information were sent to research-focused charities via the Charities Research Involvement Group – an organisation dedicated to supporting PPI in health research [18]. It was hypothesised that the collaborative aim of this group would result in a greater number of resources being shared than if individual organisations were to be indiscriminately contacted directly. Approaching a collective also allowed for a wide range of organisations to be contacted in an efficient manner.

#### Published materials

Abstracts of articles returned through the database searches were reviewed to assess suitability for inclusion in the results by EP. Those deemed to meet at least two of the inclusion criteria stated above were reviewed in full. Reference lists of the most relevant articles were checked to identify any additional relevant materials using the same approach of abstract review.

#### Unpublished materials

CTUs that responded to the request for information sent by the UKCRC and charities involved in the Charities Research Involvement Group (comprising a membership of 12 CTUs [19]) were invited to discuss their materials and methods by phone or email or to provide electronic copies of their resources directly. Any materials referenced in the documents that were not captured in the literature search were reviewed in light of the inclusion criteria above.

### Workshop

**Fig. 1:**
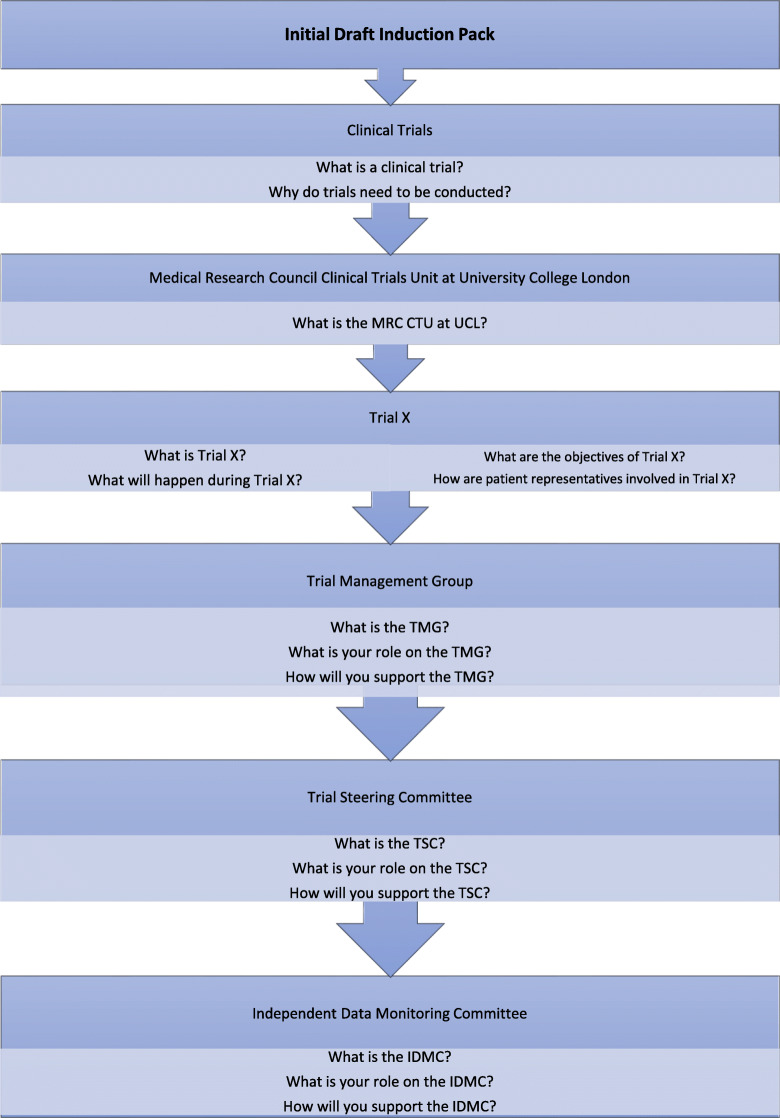
Sections of the initial draft Induction Pack presented to the workshop attendees

Fourteen PPI Contributors were invited to a morning workshop held in July 2018 at the MRC CTU at UCL. A draft Induction Pack, created from the most common themes that had emerged from the literature reviews, was circulated to those attending in advance. Themes were selected based on the frequency in which they were covered in the induction materials gathered from the literature search, with multiple mentions in the same material each counted. An assumption was made that the themes that occurred most frequently could be considered to be of most use and interest to PPI Contributors, at least in the view of the researchers creating the materials. Figure 1 contains the sections for the initial draft Induction Pack. The attendees were divided into two separate groups. One type of committee was considered by each group, based on the experience of each individual PPI Contributor so as to benefit from direct expertise and first-hand knowledge. Only one attendee had experience of IDMC membership therefore feedback was collected by email following the workshop.

Discussions were facilitated by one PPI Contributor from the MRC CTU at UCL PPI Group and one member of MRC CTU staff, supported by a student note-taker. Attendees were informed that the draft Induction Pack was open to as much change as they deemed necessary.

### Individual review

PPI Contributors were offered the opportunity to conduct a second, independent review of the final draft of the Induction Pack which incorporated the feedback from the workshop and used academic theory on adult learning to guide the presentation and ordering of information. The document was shared via email and comments were recorded and submitted via tracked changes.

### Induction pack Development

**Fig. 2:**
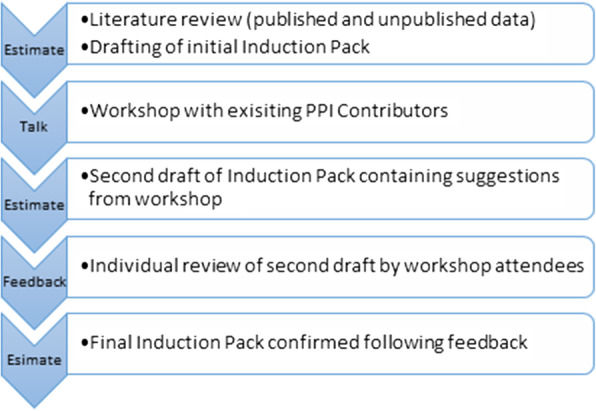
Amended Talk-Estimate-Talk Delphi approach to PPI Contributor feedback on the induction pack

So as to benefit from group, rather than individual, decision making regarding the contents and structure of the Induction Pack, an estimate-talk-estimate consensus method was implemented [20], with the addition of a further feedback-estimate round to complete the resource’s review process (Fig. 2). This process allowed the draft Induction Pack to be discussed in person as well as in writing, providing a choice of feedback mechanisms for the PPI Contributors and the opportunity to see that their initial comments had been taken on board for further review. 

Key themes and topics covered in the materials collected were extracted and triangulated through selective coding [21] to identify the most and least common subjects discussed on the topic of training and induction. These data informed the initial sections included in the draft Induction Pack produced for review at the PPI Workshop.

Notes taken from the workshop were coded and added to the existing data to confirm suitability of the current sections, identify missing topics felt to be of high importance, suggest language amendments and remove information not felt necessary for inclusion in the Induction Pack.

A final draft of the Induction Pack was subsequently produced, taking into consideration the vetted workshop feedback, and distributed to the workshop attendees for a second review. This was completed individually by workshop attendees and collated for review before being included in the final pack.

## Results

### Published materials

No published materials were found when using the search criteria laid out in the Methods. Several iterations of broader search terms were required before a manageable number of suitable abstracts were returned, but very few articles merited full review.

The published materials that were reviewed focused predominantly on the importance of adequate support for PPI Contributors in research. However, no detailed guidance or template was found in any of the results returned. Baxter et al. [22] conducted a systematic review of PPI training and support in 2016 which did identify some model resources. The articles reviewed focused on involvement in interview panels for new clinicians, wider health research or service reviews. None of the identified studies involved PPI Contributors on clinical trial oversight committees.

The EUPATI (European Patients’ Academy on Therapeutic Intervention) overview [23] gave a description of the programme and highlighted their toolbox of existing training materials but did not provide any critique of the training [24, 25]. Template training programmes are available for researchers under the title “mini-course starter kits” which group together existing resources from within the toolkit to cover certain topics, including IDMCs and TSCs^2^. These are, however, starting points for researchers to adapt and make relevant for their specific trial and parts of the course (e.g. quizzes for each section) are yet to be developed.

Of note, Bagley et al. [26] highlighted the lack of an induction pack for new PPI Contributors on trial oversight committees in Phase 2 of the creation of their toolkit for PPI in trials. Their finding reinforced the necessity of this piece of research; the existing resources identified by Bagley were reviewed and added as additional data for analysis.

### Unpublished materials

Existing induction materials from five CTUs and three research charities were provided or discussed in addition to the existing MRC CTU at UCL Induction Pack. The topics covered in these materials are summarised in Table 2. In addition, two other CTUs responded to say they did not currently have any localised induction materials but planned to create such resources in the near future. No organisation refused to share their documents.

**Table 2:** Unpublished training material subjects

Theme/Topic	Frequency
Role of a PPI Contributor	11
Explanation of what an oversight committee is	8
Number of meetings to attend	8
Who to contact for more information/support	7
Background to the specific trial under review	6
Key qualities of a PPI Contributor	6
Description of the role of each committee	5
Activity required between meetings	5
Confidentiality	5
Payment/expenses	5
Explanation of what PPI is	4
Non-attendance of meetings	4
Who makes up the membership of the committee	4
Evaluation of involvement (feedback/review)	4
Examples of activity of Contributors may undertake	3
Background to clinical trials	3
What happens at a committee meeting	3
Duration of the committee membership	3
Terms of Reference for the committee	3
Specific training to be completed	3
Trial timeline (from idea conception to results published)	2
Implications of involvement on studies (benefits/tax codes etc.)	2
Location of committee meetings	2
Training predominantly conducted as a face-to-face discussion	2
Importance of Plain English	1
Background to trial design	1
Common challenges to research	1
Developing a proposal	1
Getting your voice heard at meetings	1
Resignation from the committee	1
Safety checks to be completed (e.g. DBS)	1
Application process for committee membership	1
Explanation of what a CTU is	1
Glossary of terms	1

### Workshop

The half-day workshop for review of the draft Induction Pack was attended by twelve of the fourteen PPI Contributors invited, all of whom have experience of sitting on trial oversight committees at the MRC CTU. All attendees supported the need for the creation of an improved Induction Pack and discussions from each of the two separate groups provided similar feedback (Table 3).

**Table 3:** Feedback from workshop

To Improve	To Include	To Consider
**Terminology** •Use “plain English” not “lay language” •Provide definitions of key terms •Make language used more consistent throughout (e.g. standard of care vs current treatment)	**Additional Sections** •Glossary/jargon buster •Signposting for further information (in person or via additional resources) •Supporting/encouraging the distribution of results and implementation into standard practice where possible •Advice on getting your voice heard in meetings/teleconferences •Implications of committee membership on personal benefits/taxes (in relation to honorariums) •Encouragement to bring other skills to the group rather than “just” a PPI Contributor •Breakdown of the individual members of the committees and whether they’re independent or not •Duration of the committee membership, anticipated workload & how to leave the committee •Access to the protocol and PIS/ICF •Information on what research or trials will have been conducted in the lead up to this study •Why PPI is important •Confidentiality and how to deal with the media	**Terminology** •"Patient Representative” is an inappropriate title for some situations (e.g. people with HIV or parents/carers of children) •Clarification of the meaning of “involvement” and “engagement”
**Formatting** •Ensure consistency of visual style •Re-ordering of the sections: introduction to trials, then the CTU, then trial specific information		**Pre-Requisites** •Does the CTU require PPI Contributors to have GCP training? •Indemnity insurance - are PPI Contributors covered by the CTU’s policies?
**Language Choices** •Give clearer description of remit of this role to reduce uncertainty (e.g. remove “not expected to … “) •Sentence length is too long for plain English in some sections •Academic language is still used throughout - needs to be simplified		**Layout** •"Pick and Mix” design where trial teams can choose which sections to include •Highlight “essential” and “optional” sections
**Existing Sections** •Detail the additional visit/testing burden on participants on the study compared to standard of care •Give more details of the aims and objectives of the study under consideration in the trial specific section •Provide links to existing documents and resources rather than creating new material •Include examples of the tasks PPI Contributors may be asked to participate in •Clarify which committees see raw data broken down by treatment group		**Future Additional Resources** •Trial-specific guidance on the role and remit of each committee, to be agreed by all members of the committee to ensure consistency throughout the CTU •Future iterations to include multimedia to make the document more accessible (e.g. to those with visual impairments) •Implementation of a “Scientific Mentor” role to provide ongoing support regarding clinical aspects of the study •Similar induction pack to be created for researchers to assist them in supporting their PPI Contributors •Guidance for pre-trial groups such as protocol review boards
	**Clarification** •Differentiation between the role and remit of each oversight committee •Whether PPI Contributors have voting rights and how they are weighted compared to the other members •Involvement outside of the committee: may be asked to attend conferences to discuss the study •Whether Contributors can join multiple committees	
		**General** •Advise trial teams that a member of the PPI Group reviews the document completed by the Study Team before it’s distributed to PPI Contributors •Make it clear to investigators that the acceptable levels of risk taken by patients will vary from group to group •Emphasise that the PPI Contributor’s role is to focus on patient safety and wellbeing •Highlight that the role may adapt and change over time as the study progresses •Integrate the NIHR National Standards for PPI
		**Format** •Bullet points make the text easy to read •It is good to have clear sections marked for trial teams to add in specific data to personalise the pack •Keep the pack as short as possible to avoid overwhelming but provide signposting to additional resources

The importance of using plain English and short paragraphs for ease of reading was emphasised repeatedly. The differences between the three committees were not always clear to the PPI Contributors, despite many of them having been members of different committees themselves. It was acknowledged that a large amount of information and resources on the wider topic of medical research is already in the public domain, often having been created by patient support groups or charities, which could be used to supplement the Induction Pack. The workshop attendees felt that it was important to make use of these existing resources rather than “reinventing the wheel”.

### Post-workshop review

**Fig. 3:**
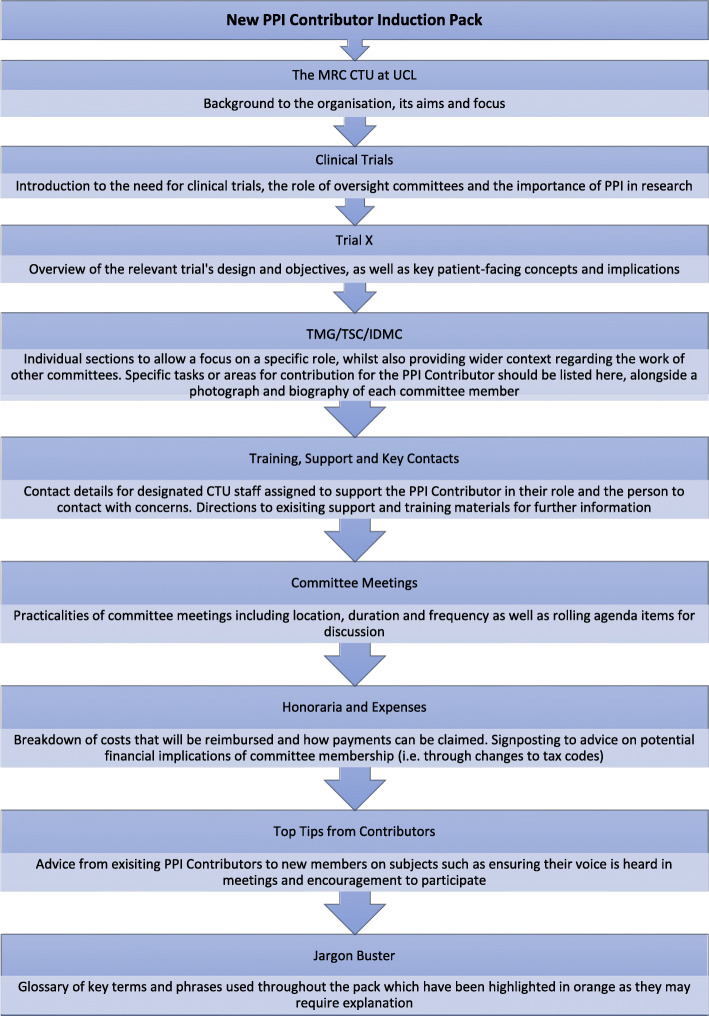
Chapter orders and summary of contents of revised induction pack

A second draft Induction Pack was created which assimilated the feedback from the workshop. The topics were reordered (shown in Fig. 3) and more attention was paid to style and format, as well as the inclusion of additional key topics and sign-posting to existing materials. The re-ordering provides a more layered approach to the information presented and allows for new knowledge to be built upon an understanding of earlier concepts [27].

Guidance notes for members of trial teams who will be completing the template were added to explain what trial-specific information needs to be provided to personalise the resource. These notes have been written in a different colour and font to distinguish them from the PPI Contributor facing information and trial teams are instructed to delete these notes before the Induction Pack is shared. The Methods and Ethics & Disseminations sections of the SPIRIT Guidelines were additionally used to guide and refine the chapter choices [28] to make completion of study-specific information as simple as possible. An assumption has been made that trial teams would be very familiar with what information is expected to be provided under these key sections.

### Individual review

Feedback on the second draft Induction Pack was received from five of the workshop attendees. The majority of the comments focused on the language used and suggested shortening sentences and using more positive and lay-friendly terms. The advice to trial teams was expanded to provide further data or information on the study’s design that was felt by the workshop attendees to be useful to new PPI Contributors.

More general feedback was also received which did not focus on the Induction Pack itself, but the training and membership of PPI Contributors on oversight committees in general. Such comments included:

- Questioning whether the selection of a new PPI Contributor should consider the breadth of knowledge of the disease under study held by the candidate to evaluate whether they could represent or convey the views of the wider patient population beyond their own personal lived experience
- Highlighting that the majority of the guidance focuses on committees for trials that have already been designed and gained funding. Therefore, it is not suitable for PPI Contributors assisting with regulatory approvals applications and initial protocol writing
- Whether un-equal voting rights between researchers and PPI Contributors should be permitted as this could impair the ability of PPI Contributors to make meaningful changes to the trial
- Encouraging the use of PPI impact assessment sheets which can be completed to track PPI activity and successes on oversight committees
- Details of the law around informed consent
- Creating a Top Tips for Researchers equivalent document
- Advising on the number of PPI Contributors that should be assigned for each committee

## Discussion

This study has found that, despite the uniformity of large aspects of the clinical trial field imposed by regulatory approval processes, international and national standards, and reporting guidelines, there is very little consistency in how patients and the public are inducted onto trial oversight committees. The lack of published materials on PPI Contributors’ training and involvement in oversight committees has allowed the creation of the Induction Pack template presented here to be very organic and predominantly influenced by direct involvement of experienced PPI Contributors. Workshops with focus groups, such as the one involved in this research, provide primary data from experts on the topic at hand.

Since all the attendees at the Induction Pack workshop had first-hand experience of sitting on trial oversight committees, their opinions and experiences provided plenty of data and ideas for analysis which could not be collected from the literature. The pack is therefore representative of the personal experiences of committee members with experience in the field and provides a high degree of ‘truth value’, or credibility, to be trusted [29, 30]. The trustworthiness of the qualitative data collected is further supported by the consistency between the notes taken from the two separate workshop groups.

Whilst different note-takers were used for each of the discussions, neither student disclosed any preconceived assumptions regarding the importance, or otherwise, of any particular topic to be discussed and therefore their records have been considered neutral representations of the conversations held. Further neutrality and completeness would have been achieved had audio recording transcriptions been used for analysis [31]. However, the use of experienced PPI Contributors rather than researchers as the primary facilitators for the focus groups reduced the feeling of distance between the attendees and the discussion’s leader, thereby providing a more neutral environment and partnership [29, 32].

### Choice of chapter topics

The methodology employed for the collection and analysis of data was carefully considered to ensure the Induction Pack’s applicability and transferability to a wider audience. Coding of both the quantitative literature data and qualitative workshop notes was conducted using the principles of grounded theory [21]: rather than looking to prove or disprove an existing theory using evidence already collected, data was reviewed alongside its collection with the aim of creating new ‘theory’. For example, as this research project commenced with the pre-defined goal of creating an Induction Pack, focused selective coding of data related to the topic facilitated the identification of key themes and issues for inclusion in the Induction Pack. It was through this analysis, and the recurrence of key categories, that the final topics for the Induction Pack were chosen therein creating new ‘theory’ to support the actions taken.

Ideas or comments that were not recurrent, or that could not be grouped into an existing category, or that fell outside the project’s predefined scope were not included in the final resource. The need for complementary guidance for researchers to support new PPI Contributors was raised on several occasions, as was the anticipated benefit of additional ongoing support mechanisms within CTUs for PPI Contributors. Had true grounded theory methodology been implemented – whereby a topic to focus upon is found from the emergent data, rather than seeking evidence related to an idea – these data and concerns might have been further explored. Other research has highlighted the need for improvements in the working relationships between PPI Contributors and academics [33]. However, the objective of this project had been set in advance and there was not scope to explore these ideas further. A potential limitation of this research is highlighted herein, suggesting that wider, or greater, issues of concern to PPI Contributors on oversight committees may have been missed as all analysis had a specific objective in mind. Future iterations could involve a more blue-sky approach with wider objectives.

Two further topics which were relevant to the final objective posed difficulties when deciding whether to include them in the Induction Pack.

The first topic concerned payments for involvement in oversight committees, which has previously been identified as a difficult subject to cover in PPI guidance because it may lead to feelings of inequality of perceived value amongst members [13]. As members of some oversight committees will be attending meetings as part of their salaried role, the difference in reimbursement for time between a CTU staff member and a PPI Contributor receiving an honorarium can be considerable. However, guidance from the National Institute for Health Research’s (NIHR) INVOLVE provides clear instruction advocating discussion and agreement of any reimbursement of expenses or payments in advance of PPI activity taking place [34]. The MRC CTU at UCL also has an in-house costing template for all PPI activity to ensure fair recompense for PPI Contributors at all stages of involvement. This topic was, therefore, included in the final Induction Pack.

The second contentious topic was whether to include information on how to withdraw from being a PPI Contributor on a committee. Opinion was split between whether providing this information could discourage new members from fully committing to long-term membership, or whether withholding these details would amount to denying the PPI Contributor the opportunity to make a fully informed decision about their participation. Negative phrasing, or priming, has been shown to produce more adverse responses to questioning [35] which supports the concerns raised at the workshop.

A compromise was reached in which explicit information was written explaining who to speak to with concerns or difficulties in fulfilling the role of PPI Contributor, on the condition that this nominated PPI Lead would, if asked, provide all the required information on how to resign from the committee. This avoids negatively framing the commitment desired from PPI Contributors without actively withholding information. As only one piece of induction material provided through the unpublished resources discussed withdrawal from a committee, it was decided that this information is not considered a requisite topic to be covered in an Induction Pack but that CTUs should have information on the processes available to PPI Contributors if requested.

### Underpinning theory

**Fig. 4:**
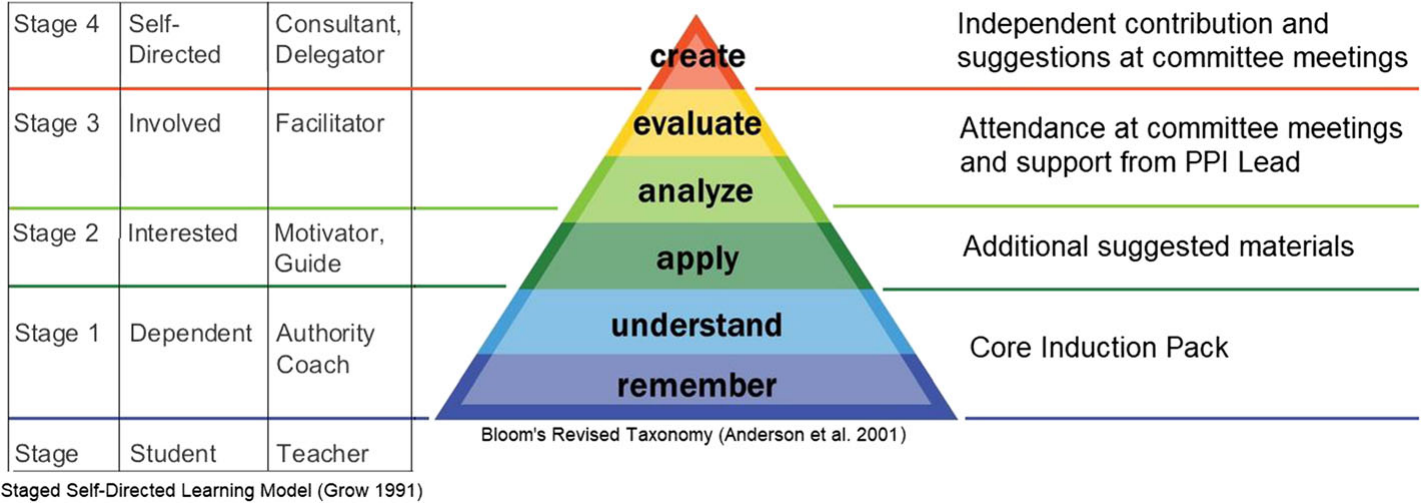
Application of Grow and Anderson theory to PPI Contributor training and how the induction pack supports active engagement in committees

It has been argued that the formal training of PPI Contributors negates the true lay perspective of a patient, carer or member of the public involved in clinical research. Ives et al. [36] claim that untrained representatives of patient groups can make substantial, specialist contributions before and after clinical research is performed (for example when applying for funding or disseminating results). However, the skills and knowledge needed to support and oversee the conduct of research requires a level of training that would minimise the ‘outsider’ or ‘lay’ perspective. This paper, however, contradicts Ives’ argument and demonstrates that, as shown in Fig. 4 using a visual representation of Anderson’s revision of Bloom’s taxonomy [27] and Grow’s Staged Self-Directed Learning [37], the thorough induction and training of PPI Contributors is essential in providing the lower levels of topic understanding required to critically appraise study information and evaluate the data from a patient’s perspective. Comprehension of jargon and research processes is vital if a PPI Contributor is to provide insight and suggestions that would maintain medical and scientific integrity [38].

In the production of this material, an assumption has been made that the reader of the Induction Pack is interested in understanding research concepts. Nevertheless, it was strongly stressed at the workshop that the entire document must be written in plain English as this could be the first exposure to technical language. This view is supported by the fact that almost 44% of the British adult population have a maximum reading comprehension of Level 1 or below which equates to that of a 12–13 year old [39].

Accessibility of the Induction Pack has been enhanced through methods such as varying the layout of the text, using boxes to highlight key concepts and writing in short, active sentences in accordance with guidance issued by the Plain English Campaign [40]. Educational materials that have been created in collaboration with learners have been found to be more successful for self-guided learning [41] therefore the re-review of the resource by the workshop attendees provides assurance of the relevance of the document to its intended audience. An active effort has been made to avoid overwhelming new PPI Contributors with information, as this is a known deterrent for successful PPI [42]. The information layering and staged approach to learning allows the speed at which the topic is explored to be specific to each individual.

### Limitations

Although this research has gathered resources and information from a range of settings for primary and secondary research, including both published and grey literature, it has limitations.

Only three examples of induction materials were received from research charities, and this gave a limited view of what materials are currently in use. However, it is worth noting that most charities do not manage or coordinate clinical trials themselves: they do not have the clinical governance structure to oversee what could be complex interventions and therefore perform more patient-focused research rather than trials. The organisations approached may not have felt that they had relevant information to share. A wider representation of CTUs would also have added to the baseline knowledge that fed into this project but, considering that most of those who did respond sit on the UKCRC CTU PPI Sub-Group, it could be presumed that the documents provided came from units with the most interest in PPI and therefore the most developed resources.

Additionally, the success of the Induction Pack will be dependent upon trial teams completing the template to a high standard and distributing it to their PPI Contributors. Advice is provided to researchers to guide the completion of study-specific details; however, all additional information will still need to be written in plain English to ensure it is suitable for its audience.

Finally, the scope of this project did not allow for evaluation and monitoring of real-life use of the Induction Pack for new PPI Contributors. We have therefore not been able to assess its practicality and utility for research teams or inductees. However, as no data was collected on PPI Contributor or research satisfaction using the previous MRC CTU at UCL’s induction pack, there would have been no data against which to compare the use of the resource created.

### Relevance of the induction pack template

The template created from this research has filled a gap in training resources for new PPI Contributors and has been made available to other CTUs to personalise and implement for their own studies and settings. This resource is not intended to form a comprehensive training programme, but to provide a starting point for new PPI Contributors and the support of PPI Teams within CTUs or research organisations. The value of this role on trial oversight committees has thus far been underreported. It is hoped that through providing a template Induction Pack for CTUs more researchers will be encouraged to increase their PPI activity at committee level and track and evaluate the impact that comprehensively trained PPI Contributors can have on trial governance.

## Conclusion

To our knowledge, this is the first study to identify the requisite elements of an Induction Pack for PPI Contributors sitting on trial oversight committees. It provides a starting point for documenting the creation of evidence-based training materials which have been shown to be lacking in this field [26].

The Induction Pack created as a result of this research is supported by current practice evidenced through published and unpublished literature alongside PPI review. The template demonstrates the wide range of topics to be considered essential when training lay members to join highly specialised committees which discuss very niche and jargonistic topics. Whilst the final resource created refers to clinical trials, we believe that other researchers could make small adaptations to the Induction Pack for use in other trials, such as within social care.

Further research should be conducted to create complementary guidance for researchers welcoming new PPI Contributors to trial oversight committees. This would support the development of more productive working relationships between PPI Contributors and researchers which has been identified as a top priority for future methodological research into PPI [33]. The role of PPI Contributors on oversight committees is under-represented in the literature and further research (such as the expansion of Daykin et al.’s study of relationships in oversight committees [43]) is eagerly anticipated.

The final Induction Pack created can be found online at https://www.ctu.mrc.ac.uk/patients-public/patient-public-involvement-resources/papers-guidance-templates/

## Data Availability

The induction pack created is included in this published article. No datasets were generated or analysed.

## Abbreviations

CTU: Clinical Trials Unit
EUPATI: European Patients’ Academy on Therapeutic Intervention
IDMC: Independent Data Monitoring Committee
MRC CTU at UCL: Medical Research Council Clinical Trials Unit at University College London
NIHR: National Institute for Health Research
PPI: Patient and Public Involvement
TMG: Trial Management Group
TSC: Trial Steering Committee
UKCRC CTU: United Kingdom Clinical Research Collaboration’s Registered Clinical Trial Units Network

## Footnotes

1 We have used the term ‘PPI contributor’ to describe the patients, carers, community members and members of the public who get involved in our research.

2 The TSC starter kit was unlocatable on the website and therefore could not be reviewed. It is, however, referenced several times throughout the website. It has been assumed that a link or landing page is missing

## References

[1] European Medicines Agency (2005). Guideline on Data Monitoring Committee.

[2] Conroy EJ, Harman NL, Lane JA, Lewis SC, Murray G, Norrie J, Sydes MR, Gamble C (2015). Trial steering committees in randomised controlled trials: a survey of registered clinical trials units to establish current practice and experiences. Clin Trials.

[3] Liabo K, Boddy K, Burchmore H, Cockcroft E, Britten N (2018). Clarifying the roles of patients in research. BMJ..

[4] Richards T, Snow R, Schroter S (2016). Co-creating health: more than a dream. BMJ..

[5] Stewart R, Liabo K (2012). Involvement in research without compromising research quality. J Health Serv Res Policy.

[6] Richards T, Montori VM, Godlee F, Lapsley P, Paul D (2013). Let the patient revolution begin. BMJ..

[7] NIHR. Apply for Funding [Internet]. Make a Strong Application. [cited 2020 Jan 05]. Available from: https://www.nihr.ac.uk/researchers/apply-for-funding/how-to-apply-for-project-funding/make-a-strong-application.htm

[8] UK Research and Innovation. Guides, policies, research and publications - UK Research and Innovation [Internet]. Guides, policies, research and publications. [cited 2018 Apr 29]. Available from: https://www.ukri.org/public-engagement/research-council-partners-and-public-engagement-with-research/guides-policies-research-and-publications/

[9] Diabetes UK. Patient and public involvement (PPI) in your study [Internet]. Diabetes UK. [cited 2018 Apr 29]. Available from: https://www.diabetes.org.uk/research/for-researchers/apply-for-a-grant/help-with-involving-participants

[10] British Geriatrics Society. Patient and Public Involvement in Research [Internet]. Patient and Public Involvement in Research. [cited 2018 Apr 29]. Available from: https://www.bgs.org.uk/resources/patient-and-public-involvement-in-research

[11] The European Parliament and the Council of the European Union. Clinical Trial Regulation EU No. 536/2014 [Internet]. Clinical Trial Regulation. 2014 [cited 2018 Apr 26]. Available from: https://ec.europa.eu/health//sites/health/files/files/eudralex/vol-1/reg_2014_536/reg_2014_536_en.pdf

[12] UK Public Involvement Standards Development Partnership. UK Standards for Public Involvement [internet] UK Standards for Public Involvement [cited 2020 Jan 05]. Available from: https://sites.google.com/nihr.ac.uk/pi-standards/standards

[13] Lockey R, Sitzia J, Gillingham T, Millyard J, Miller C, Ahmed S (2004). Training for service user involvement in health and social care research: final report. INVOLVE.

[14] Nicholson A, Daykin A, Macefield R, McCann S, Shorter G, Sydes M, Gamble C, Shaw A, Lane JA (2015). Enhancing public involvement in trial oversight committees through qualitative research with eight trials facing challenges. Trials..

[15] MRC Clinical Trials Unit. Trial design and management [Internet]. Medical Research Council Clinical Trials Unit: Resources. [cited 2018 Aug 27]. Available from: http://www.ctu.mrc.ac.uk/resources/trial_design_and_management/

[16] UK Clinical Research Collaboration Registered Clinical Trials Units Network. Homepage [Internet]. UKCRC CTU Network. [cited 2018 Sep 8]. Available from: https://www.ukcrc-ctu.org.uk/

[17] UK Clinical Research Collaboration Registered Clinical Trials Units Network. Supplementary Terms of Reference Patient and Public Involvement & Engagement (PPI&E) Task and Finish Group [Internet]. 2017 [cited 2018 Aug 12]. Available from: https://cdn.ymaws.com/www.ukcrc-ctu.org.uk/resource/resmgr/sub_groups_general/PPI_TOR_Final_27.02.17.pdf

[18] Shared Learning Group on Involvement. Homepage [Internet]. Shared Learning Group on Involvement. [cited 2018 Sep 8]. Available from: http://slginvolvement.org.uk/

[19] UKCRC Registered Clinical Trials Units Network. Task and Finish Groups [Internet]. UKCRC CTU [cited 2020 Nov 1]. Available from: https://www.ukcrc-ctu.org.uk/page/TFGroups

[20] Gustafson DH, Shukla RK, Delbecq A, Walster GW (1973). A comparative study of differences in subjective likelihood estimates made by individuals, interacting groups, Delphi groups, and nominal groups. Organ Behav Hum Perform.

[21] Glaser B, Strauss A (1967). Discovery of grounded theory : strategies for qualitative Research.

[22] Baxter S, Clowes M, Muir D, Baird W, Broadway-Parkinson A, Bennett C (2017). Supporting public involvement in interview and other panels: a systematic review. Health Expect.

[23] EUPATI. EUPATI Training Course [Internet]. EUPATI. [cited 2018 Aug 12]. Available from: https://www.eupati.eu/eupati-training-course/

[24] Chakradhar S. Training on trials: Patients taught the language of drug development [Internet]. Nature Medicine. 2015 [cited 2018 May 12]. Available from: https://www.nature.com/articles/nm0315-209 10.1038/nm0315-209 25742451

[25] EUPATI. Patient education! The A to Z of medicines development [Internet]. EUPATI Toolbox. 2016 [cited 2018 Apr 29]. Available from: https://www.eupati.eu

[26] Bagley HJ, Short H, Harman NL, Hickey HR, Gamble CL, Woolfall K, Young B, Williamson PR (2016). A patient and public involvement (PPI) toolkit for meaningful and flexible involvement in clinical trials – a work in progress. Res Involv Engagem.

[27] Anderson LW, Krathwohl DR (2001). A taxonomy for learning, teaching, and assessing: a revision of Bloom’s taxonomy of educational objectives. Complete ed..

[28] Chan A-W, Tetzlaff JM, Altman DG, Dickersin K, Moher D (2013). SPIRIT 2013: new guidance for content of clinical trial protocols. Lancet.

[29] Lincoln YS, Guba EG (1985). Naturalistic inquiry.

[30] Guba EG (1981). Criteria for assessing the trustworthiness of naturalistic inquiries. ECTJ..

[31] Bertrand JT, Brown JE, Ward VM (1992). Techniques for analyzing focus group data. Eval Rev.

[32] Krefting L (1991). Rigor in qualitative Research: the assessment of trustworthiness. Am J Occup Ther.

[33] Kearney A, Williamson P, Young B, Bagley H, Gamble C, Denegri S, Muir D, Simon NA, Thomas S, Elliot JT, Bulbeck H, Crocker JC, Planner C, Vale C, Clarke M, Sprosen T, Woolfall K (2017). Priorities for methodological research on patient and public involvement in clinical trials: a modified Delphi process. Health Expect.

[34] INVOLVE. Payment and recognition for public involvement [Internet]. [cited 2018 Sep 2]. Available from: http://www.invo.org.uk/resource-centre/payment-and-recognition-for-public-involvement/

[35] Claessen FMAP, Mellema JJ, Stoop N, Lubberts B, Ring D, Poolman RW (2016). Influence of priming on patient-reported outcome measures: a randomized controlled trial. Psychosomatics..

[36] Ives J, Damery S, Redwod S (2013). PPI, paradoxes and Plato: who’s sailing the ship?. J Med Ethics.

[37] Grow GO (1991). Teaching learners to be self-directed. Adult Educ Q.

[38] Staley K (2013). There is no paradox with PPI in research. J Med Ethics.

[39] Department for Business, Innovation & Skills. 2011 Skills For Life Survey [Internet]. Department for Business, Innovation & Skills; 2012 Dec [cited 2018 Jun 2]. (BIS Research Papers). Available from: https://www.gov.uk/government/publications/2011-skills-for-life-survey

[40] Plain English Campaign. How to Write Medical Information in Plain English [Internet]. 2001 [cited 2018 Sep 4]. Available from: http://www.plainenglish.co.uk/files/medicalguide.pdf

[41] Merriam SB (2001). Andragogy and self-directed learning: pillars of adult learning theory. New Dir Adult Contin Educ.

[42] Staley K (2009). Exploring impact: public involvement in NHS, public health and social care research.

[43] Daykin A, Selman LE, Cramer H, McCann S, Shorter GW, Sydes MR (2017). ‘We all want to succeed, but we’ve also got to be realistic about what is happening’: an ethnographic study of relationships in trial oversight and their impact. Trials.

